# Evolutionary Changes on the Way to Clathrin-Mediated Endocytosis in Animals

**DOI:** 10.1093/gbe/evw028

**Published:** 2016-02-12

**Authors:** Mykola Dergai, Anton Iershov, Olga Novokhatska, Serhii Pankivskyi, Alla Rynditch

**Affiliations:** 1 Department of Functional Genomics, Institute of Molecular Biology and Genetics, NASU, Kyiv, Ukraine; 2 Department of Biosynthesis of Nucleic Acids, Institute of Molecular Biology and Genetics, NASU, Kyiv, Ukraine

**Keywords:** evolution of endocytic system, endocytic protein interaction network, scaffolding FCHO/Eps15/ITSN complex, regulation of AP2 conformational switch, clathrin-associated sorting proteins, dynamin/SNX9 complex

## Abstract

Endocytic pathways constitute an evolutionarily ancient system that significantly contributed to the eukaryotic cell architecture and to the diversity of cell type–specific functions and signaling cascades, in particular of metazoans. Here we used comparative proteomic studies to analyze the universal internalization route in eukaryotes, clathrin-mediated endocytosis (CME), to address the issues of how this system evolved and what are its specific features. Among 35 proteins crucially required for animal CME, we identified a subset of 22 proteins common to major eukaryotic branches and 13 gradually acquired during evolution. Based on exploration of structure–function relationship between conserved homologs in sister, distantly related and early diverged branches, we identified novel features acquired during evolution of endocytic proteins on the way to animals: Elaborated way of cargo recruitment by multiple sorting proteins, structural changes in the core endocytic complex AP2, the emergence of the Fer/Cip4 homology domain-only protein/epidermal growth factor receptor substrate 15/intersectin functional complex as an additional interaction hub and activator of AP2, as well as changes in late endocytic stages due to recruitment of dynamin/sorting nexin 9 complex and involvement of the actin polymerization machinery. The evolutionary reconstruction showed the basis of the CME process and its subsequent step-by-step development. Documented changes imply more precise regulation of the pathway, as well as CME specialization for the uptake of specific cargoes and cell type-specific functions.

## Introduction

One of the principal features of the eukaryotic cell that distinguishes it from prokaryotic cell is complex endocytic system. According to the current view, the role of this system in evolution lies in its impact on actuation of the eukaryotic cell plan (Sigismund et al. 2012). Furthermore, during eukaryote evolution endocytic system appeared as a platform and central organizer for such cellular pathways as intracellular transport and signal transduction. In animal cells endocytosis is essential for internalization of activated receptors and regulation of signaling, maintenance of plasma membrane (PM) homeostasis, as well as cell type–specific functions ranging from synaptic transmission to antigen presentation (Sigismund et al. 2012).

Clathrin-mediated endocytosis (CME) is a universal internalization route in eukaryotes (Samaj et al. 2005; Macro et al. 2012; Adung’a et al. 2013; Field et al. 2007) and the best-studied endocytic pathway to date. The biochemistry of CME is much better understood in animals than in other eukaryotes (Kirchhausen et al. 2014). Initiation of CME starts with the recruitment of the adaptor protein 2 complex (AP2) complex and clathrin chains to the PM (Cocucci et al. 2012). Specific cargoes designated for internalization via CME are recruited to the nascent clathrin-coated pits (CCPs) either by AP2 or clathrin-associated sorting proteins (CLASPs) (Traub and Bonifacino 2013). Transition of AP2 to the open conformation is required to dock clathrin to molecular cargoes at PM and is regulated via interactions with PM, cargo (Owen et al. 2004), and the FEI functional complex (Hollopeter et al. 2014; Umasankar et al. 2014). Components of the FEI complex—FCHO (Fer/Cip4 homology domain-only protein), Eps15 (epidermal growth factor receptor substrate 15), and ITSN (intersectin)—arrive early to the places of CCP formation and are important for assembly of nascent pits (Henne et al. 2010) and their maturation (Loerke et al. 2009; Henne et al. 2010). Increase in CCP membrane curvature is mediated by membrane-bending accessory proteins and clathrin polymerization. The clathrin lattice around the endocytic membrane supports its deformation and defines the size of the vesicle (Kirchhausen et al. 2014). Late CME stages that precede vesicle fission require the sorting nexin 9 (SNX9) protein for conversion of CCP to a deeply invaginated structure and remodeling of the vesicle neck (Posor et al. 2013). The GTPase dynamin (DNM) forms a ring at the neck of CCV and mediates vesicle fission from the PM. Late stages of invagination and fission are supported by the network of actin filaments (McMahon and Boucrot 2011; Kirchhausen et al. 2014). After vesicle fission, the clathrin lattice is disassembled by uncoating proteins providing recycled proteins for the next cycles of endocytosis. CME is highly integrated with other cellular processes, in particular signal transduction, protein sorting, secretion, and degradation (Traub 2009; McMahon and Boucrot 2011).

Despite extensive investigation of CME over the last three decades, a number of fundamental questions still remain. What is the very basis of CME that could be observed in extant eukaryotes? To what extent are the CME of animals and yeast related regarding the latter as widely used model for CME studies? How was CME linked to other cellular processes, in particular signaling? These questions could be answered by reconstruction of CME evolution. Advances over the past decade in genome sequencing have led to the elaboration of comparative and evolutionary studies in molecular and cell biology. The availability of complete genomes and proteomes allowed the investigation of evolutionary changes in proteins and their families; however, what is more intriguing is the possibility of evolutionary reconstructions of cellular pathways and systems.

To date, several reports described the evolutionary histories of individual endocytic proteins (Wakeham et al. 2005; Traub 2009; De Craene et al. 2012; Purkanti and Thattai 2015) or the distribution of several CME components in eukaryotes (Field et al. 2007). Yet, however, reconstruction of evolution of the CME process is still missing. In this work, comparative proteomic studies were used to analyze how the molecular machinery of CME was modified during eukaryote evolution on the way to animals. We searched for “waves of emergence,” functional subsets of proteins specific for large taxonomic groups, and investigated how the molecular basis for certain endocytic features was developed. To this end phylogenetic analysis was combined with functional data reported previously.

Here we provide data on the evolutionary history of CME typical of animals. We traced the emergence of essential animal endocytic proteins and their modifications during evolution of eukaryotes from the last eukaryotic common ancestor (LECA) to metazoans. The role of these evolutionary events is discussed in relation to their possible impact on the mechanism of CME, thereby shaping endocytosis to that observed in extant animals, particularly in mammals.

## Materials and Methods

### Search for Homologs of Endocytic Proteins

One hundred fifty-one reference proteomes of eukaryotic organisms were fetched from the Broad Institute (www.broadinstitute.org, last accessed August 16, 2015) databases (*Fonticula alba*, *Thecamonas trahens*, *Sphaeroforma arctica*, *Monosiga brevicolis*, *Capsaspora owczarzaki*) and UniProt (http://www.uniprot.org/, last accessed August 16, 2015) (the other organisms). To find potential homologs of animal endocytic proteins, we first constructed Hidden Markov Models (HMMs) for each of the 35 endocytic proteins using HMMER (Finn et al. 2011) on the basis of the multiple sequence alignment produced by Clustal Omega (Sievers et al. 2011). We used sampling of four model organisms, *Ciona intestinalis*, *Caenorhabditis elegans*, *Drosophila melanogaster*, and *Homo sapiens*, that represent diversity in the metazoan group. The HMMs obtained were used as a query to search for homologous endocytic proteins in the proteomes with a sequence inclusion *e*-value threshold of 0.01 and domain inclusion threshold value of 0.01 as recommended by HMMER. Then we checked the domain architecture of the forward search hits. Protein domains were predicted with HMMER using curated domain models from Pfam 28.0 release (Finn et al. 2014) or Prosite (http://prosite.expasy.org/, last accessed September 4, 2015). Coiled-coil regions were predicted with ncoils (Lupas et al. 1991). We selected only the hits with domain architecture typical of animal proteins that scored first corresponding to human homologs of the endocytic proteins during reciprocal search against the human proteome. In the case of distant proteins, that is, clathrin in Euglenozoa, we selected the forward search hits that had typical domain architectures.

Accession numbers of the homologs identified for each endocytic component throughout the eukaryotes together with *e*-values for the forward HMMER search for these hits are listed in supplementary table S1, Supplementary Material online. Prediction and analysis were automated using Python scripts that are available online (http://github.com/yklsorok/prot_evol_toolkit).

### Prediction of Protein Interaction Motifs

Motifs that potentially bind core endocytic proteins were predicted using regular expressions: DP[WF]|F.D.F|WV.F|F.F.[FL] for AP2 binding (Praefcke et al. 2004), L[FILMV].[FILMV][DE]|L[FILMV].[DE][FILMV] for clathrin binding (Dell’Angelica 2001; Lafer 2002), and asparagine-proline-phenylalanine motif (NPF) for Eps15 homology (EH) domain binding. For all motifs predicted in CLASPs, we counted the occurrence of each motif in the phylum as the number of organisms that have the motif in the corresponding protein divided by the total number of organisms in the phylum. We also counted the median occurrence of each motif in the phylum as the median of motif counts for all proteins in the phylum that contain the corresponding motif (supplementary table S8, Supplementary Material online). We took into account only motifs in the protein “tails,” C-terminal regions lacking globular domains, and in the case of stonin (STON) we counted the motifs in the N-terminal region.

To predict AP2-activating region (APA) motifs, we generated the HMM profile using the APA motifs of *H. sapiens*, *Mus musculus*, and *Ca. elegans* FCHO homologs that were experimentally shown to bind AP2 (Hollopeter et al. 2014) and performed a search in proteomes of eukaryotes using HMMer.

### Generation of the CME Protein Interaction Network

Binary protein–protein interactions between CME components were obtained from the BioGrid (http://thebiogrid.org/, last accessed June 8, 2015) and MINT (http://mint.bio.uniroma2.it/mint/, last accessed June 8, 2015) databases. The binding data obtained were filtered to remove those resulting from high throughput analyses, colocalizations, cofractionations, and two-hybrid screens. Therefore only binding of proteins detected by affinity purification of protein complexes, protein-fragment complementation assays, reconstituted protein complexes *in vitro*, Far Western blotting, and biochemical activity was considered. The protein-interaction network of CME was visualized with Cytoscape (www.cytoscape.org/, last accessed December 1, 2014).

### Tree Construction and Sequence Analysis

Multiple sequence alignments were generated by the Clustal Omega algorithm. Maximum-likelihood analysis was performed using the RaxML 8.1.3 software (Stamatakis 2014) with 100 bootstrap replicates using the computational cluster of the Institute of Molecular Biology and Genetics NASU (Salnikov et al. 2009). Phylogenetic trees were visualized by Dendroscope 3 (http://dendroscope.org, last accessed October 1, 2014) (Huson and Scornavacca 2012). Sequence logos were generated with WebLogo application (http://weblogo.berkeley.edu, last accessed October 2, 2015) (Crooks et al. 2004). Visualization of multiple sequence alignments was performed by ESPript 3.0 (http://espript.ibcp.fr, last accessed October 2, 2015) (Robert and Gouet 2014).

### Three-Dimensional Modeling of AP2 Multimodular Complexes

Three-dimensional (3D) models of AP2 complexes of fungus and chromalveolata were obtained by Modeller (Sali and Blundell 1993) using the crystal structure of the *Rattus norvegicus* AP2 complex (PDB code: 4UQI) (Kelly et al. 2014) as template. Missing residues located at disordered loops in chains alpha, beta, and mu were generated and included in the 4UQI template structure by loop modeling as provided by Modeller. The AP2 protein sequences of *Blastocystis hominis* and *Cryptococcus cinerea* were obtained from the NCBI GenBank. Pairwise sequence alignments were produced using Clustal Omega and formatted manually according to the syntax of Modeller *.ali files. Ensembles of 20 multichain models were generated and the best models were selected by the smallest value of normalized discrete optimized protein energy score for further analysis. To check Modeller scores independently, we used the MolProbity suite (Chen et al. 2010) that allowed to perform a comprehensive analysis of protein structure, residue by residue, and calculates the score using the statistical potential and physics-based approaches. Superposition and visualization of 3D structures was performed by PyMOL (Schrödinger 2010).

## Results

### Emergence of Endocytic Components Typical of Animals and Organization of an Endocytic Protein Interaction Network

To follow transformation of CME components from LECA to metazoans, we selected 35 proteins essential for vesicle formation and cargo internalization in animals (McMahon and Boucrot 2011; Kirchhausen et al. 2014). Certainly, the list of CME proteins assembled was a reduction of the known complexity, but this approach allowed us to disclose basic CME components and their functional links escaping abundant data. Furthermore, we investigated the distribution of selected CME proteins across eukaryotes to infer the possible time of their emergence.

To address questions of emergence and evolution of CME components we used systematics recently upgraded by Cavalier-Smith group (Cavalier-Smith et al. 2014). The eukaryotic evolutionary tree was regarded as two supergroups, Bikonta and Unikonta. The first supergroup comprised Archaeplastida (plants and algae) and Chromista (stramenopiles, alveolates, rhizarians, etc.) branches as well the earliest deducible eukaryotic branches, Excavata and Euglenozoa. The second supergroup encompassed Amoebozoa (amoebas and slime molds) and Opisthokonta (fungi, animals, and related organisms), the latter of which finally gave raise to extant Metazoa. We focused our attention on representatives of Unikonta and moved along the eukaryotic tree toward animals identifying common conserved features and emerging ones. Within Unikonta there were several taxa on the way to animals: Obazoa, Opisthokonta, Holozoa, and Metazoa. Extant animals, Metazoa, together with related unicellular Choanozoa formed Holozoa group. Furthermore, Fungi, related Nucleariidae and Holozoa belonged to the Opisthokonta branch. Nicleariidae was considered close to the root of the branch. Apusozoa branched before common ancestor of Opisthokonta and together they comprised Obazoa. Comparative study of eukaryotes belonging to Bikonta and Unikonta supergroups followed by analysis of taxa of different ranks allowed us to identify emerging CME components and their features.

The search of proteins homologous to animal endocytic components was automated using Python scripts for the 151 proteomes representing all major eukaryotic branches (supplementary table S1, Supplementary Material online). We tried to balance taxonomic groups to provide maximal coverage and diversity within groups and avoid oversampling certain groups, for example, mammals over worms and therefore bias. The HMMs were constructed from sequence alignments of *Ci. intestinalis*, *Ca. elegans*, *D. melanogaster*, and *H. sapiens* endocytic proteins. Hits with the same domain architecture as in animals were run as reciprocal query against the human proteome. The criterium to assign an identified hit as a homolog of an animal protein was the requirement for it as reciprocal best hit. Best reciprocal hits representing proteins conserved within certain taxons with partially similar domain architectures were considered as putative analogs or ancestors of animal proteins (supplementary table S2, Supplementary Material online). Identified homologs of the animal endocytic proteins were further analyzed for structural features with reported functional roles.

**Fig. 1.—:**
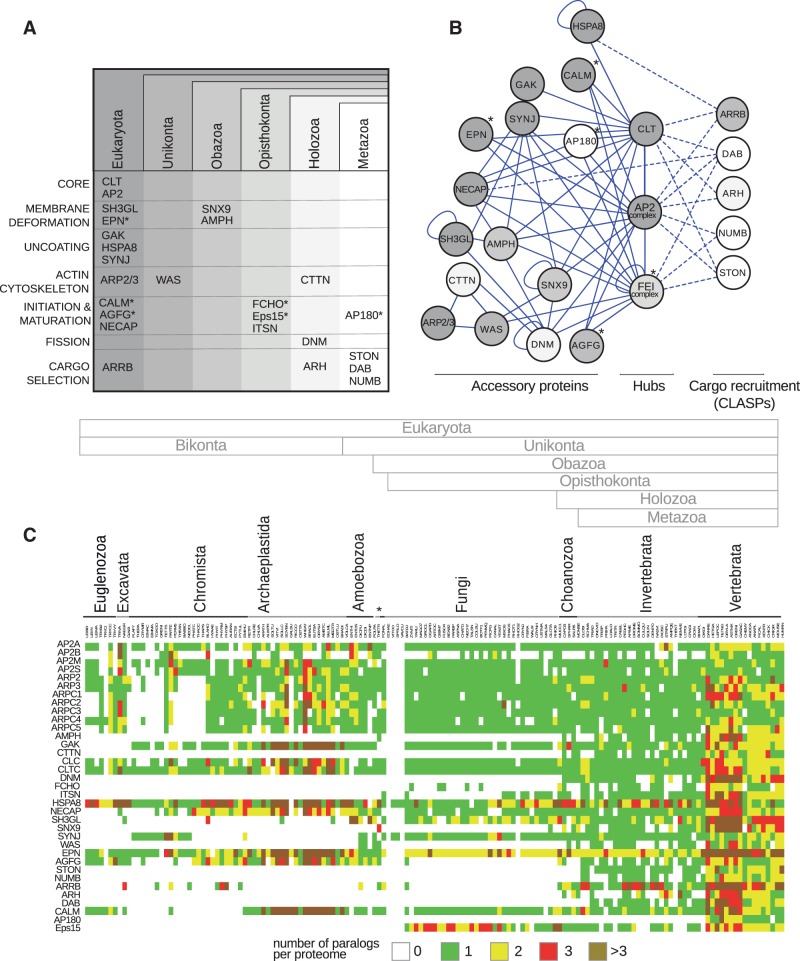
Emergence of CME components essential in animals. (*A*) Distribution across eukaryotes of the proteins involved in CME of metazoans. Selected endocytic proteins were searched with HMMer in 151 complete proteomes representing all major eukaryotic branches (supplementary table S1, Supplementary Material online). Each CME component is indicated in the evolutionary earliest eukaryotic branch where its homolog was identified. Holozoa includes Choanozoa and Metazoa; Opisthokonta consists of Holozoa and Fungi; Obazoa includes Opisthokonta and Apusozoa. Proteins are arranged according to the time of their emergence and primary known function in CME. Accessory proteins with the ability of cargo binding are indicated by asterisks. CLT includes two chains, CLC and CLTC; the AP2 complex consists of four subunits, AP2A, AP2B, AP2M, and AP2S; the ARP2/3 complex includes seven subunits: ARP2, ARP3, and ARPC1-5. (*B*) Protein interaction network of animal CME proteins. Interactions of accessory proteins (lines) and CLASPs (dashed lines) are shown. The shade of gray is consistent with the time of emergence of the endocytic component in evolution (*A*). The FEI functional complex consists of the FCHO, Eps15, and ITSN components. (*C*) Distribution of paralogs of endocytic components in proteomes representing major eukaryotic branches. Color scheme shows the number of homologs identified by reciprocal HMMer search (see Material and Methods; supplementary tables S1 and S3, Supplementary Material online). Proteins and species names are indicated and arranged from top to bottom and from left to right of heat map, respectively, in the same order as in supplementary table S3, Supplementary Material online. Asterisks denote apusomonad *Thecamonas trahens* and nucleariid *Fonticula alba*.

Our data showed that a subset of 22 CME proteins was widely distributed throughout eukaryotes (fig. 1*A*). The remaining 13 endocytic proteins were gradually incorporated into the CME machinery during evolution of eukaryotes from LECA to Metazoa. The actin cytoskeleton regulator Wiskott-Aldrich syndrome (WAS) was found in Unikonta and could link the process of actin polymerization to membrane deformation and vesicle fission. In Obazoa, two membrane-deforming proteins with BAR domains (Bin/Amphiphysin/Rvs domain) were documented, SNX9 and AMPH (amphiphysin). Distinctive features of SNX9 from other sorting nexins are the N-terminal Src homology 3 (SH3) domain that mediates protein–protein interactions and specific BAR domain, classified by Pfam service as WASP-binding BAR domain. AMPH has the same domain organization as SH3GL (SH3 domain GRB2-like) found in LECA; however, AMPH is capable of binding to endocytic hubs AP2 and clathrin as well as producing and “sensing” higher degrees of PM curvature than SH3GL (Peter et al. 2004). Later, in the Opisthokonta the FEI functional network considered as essential organizer of early CME stages in animals and fungi (Weinberg and Drubin 2012; Hollopeter et al. 2014) was documented. In this taxon, subgroup of CLASPs, endocytic proteins that recruit specific types of cargoes to nascent CCPs expanded. Late CME components, DNM and CTTN (cortactin) (Cao et al. 2003;Lundmark and Carlsson 2004), that contribute to CCV fission have been found since Holozoa (fig. 1*A*).

To conceive changes in CME upon emergence of novel components from LECA to Metazoa, we generated Protein Interaction Network (PIN) based on previously reported biochemical data deposited in the Biogrid and MINT databases (fig. 1*B*). Endocytic proteins were allocated within PIN according to their function either in vesicle formation or recruitment of a particular cargo. As a result three groups with distinct properties were observed. Endocytic accessory proteins emerged relatively early during evolution of eukaryotes and formed redundant interconnections within their group. The majority of CLASPs that represent a more recent evolutionary innovation were isolated from each other and from accessory proteins. In addition to their role in vesicle formation, the accessory proteins Eps15, EPN (epsin), CALM (clathrin assembly lymphoid myeloid leukemia protein), AP180 (clathrin coat assembly protein AP180), AGFG (Arf-GAP domain and FG repeat-containing protein), and FCHO also participated in cargo recognition (Traub and Bonifacino 2013). Interaction between these groups was mediated by three hubs: AP2, clathrin, and the FEI proteins. Therefore during evolution endocytic machinery gained group of sorting proteins and an additional endocytic interaction hub with a functional role similar to AP2 in integration accessory and sorting components.

Many CME components are represented by families of paralogous proteins in mammals. We analyzed the timing and the extent of duplication of genes encoding endocytic proteins. Paralogous expansion of these genes occurred in a common ancestor of vertebrates (fig. 1*C* and supplementary table S3, Supplementary Material online) apparently during the last whole genome duplication (Smith et al. 2013). Therefore 60% of endocytic components in vertebrates have paralogs in contrast to 12% observed in invertebrates. Noteworthy is that a tendency of decrease in the number of paralogs for each endocytic component was observed in tetrapods compared with fish.

Thus, in addition to the subset of endocytic proteins found in LECA, novel CME components were added during evolution implying modification of endocytic PIN. Since vertebrates, the majority of endocytic components have been represented by several proteins due to massive paralogous expansion of endocytic genes.

### Eps15 and ITSN Are Opisthokont-Specific Variants of Conserved Endocytic EEC Proteins

One of the first documented “waves of emergence” of endocytic components was the acquisition in opisthokonts of three functionally linked proteins, FCHO, Eps15 and ITSN, that constitute the FEI functional complex (Henne et al. 2010; Mayers et al. 2013). The adaptor proteins Eps15 and ITSN possess multiple protein-interaction domains and by hetero-oligomerization provide large scaffolding for assembly of early endocytic complexes. To trace the evolutionary history of these proteins typical of the opisthokonts, we searched for domain architectures from which Eps15 and ITSN could originate, in particular EEC, SH3-SH3, DH-PH, and DH-PH-C2 in proteomes representing major eukaryotic branches.

**Fig. 2.—:**
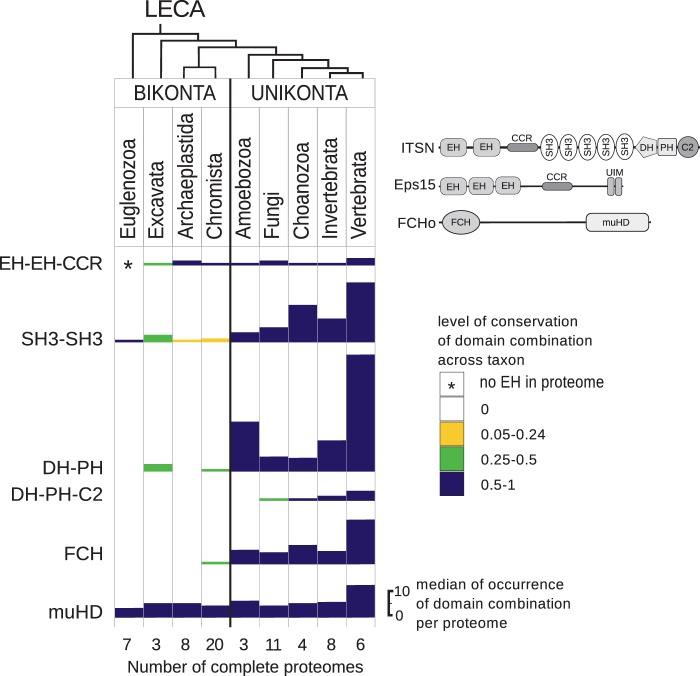
Occurrence of domain combinations typical of FEI components in proteomes of major eukaryotic branches. The level of conservation of the domain combinations across taxa is presented in different colors. Bars represent median value of occurrence of domain combinations per proteome within the indicated taxa. Relations between clades are shown above according to Cavalier-Smith et al. (2014); the number of complete proteomes investigated is indicated below.

The EEC domain architecture that represented the N-terminal region of Eps15 and ITSN was the most conserved domain combination studied throughout eukaryotes (fig. 2 and supplementary table S4, Supplementary Material online). In eukaryotes beyond opisthokonts, the EEC is a protein without additional globular domains according to the annotations of PROSITE, SMART, and Pfam. EEC proteins could be found in all major eukaryotic branches except in the Euglenozoa, a taxon that completely lacks EH domains. Low occurrence of EEC proteins in proteomes in comparison with other domain architectures studied (e.g., SH3-SH3, DH-PH) together with conservation across eukaryotes suggested specific functions for these proteins. In opisthokonts, EEC-containing proteins have specific C-terminal domains. Eps15 has ubiquitin-binding regions, ITSN has multiple SH3 domains together with a DH-PH-C2 extension, and Reps possesses a RalA binding protein domain. We assumed that the EEC proteins of primitive eukaryotes could be ancestors of EEC-containing Eps15, ITSN, and Reps molecules in animals.

**Fig. 3.—:**
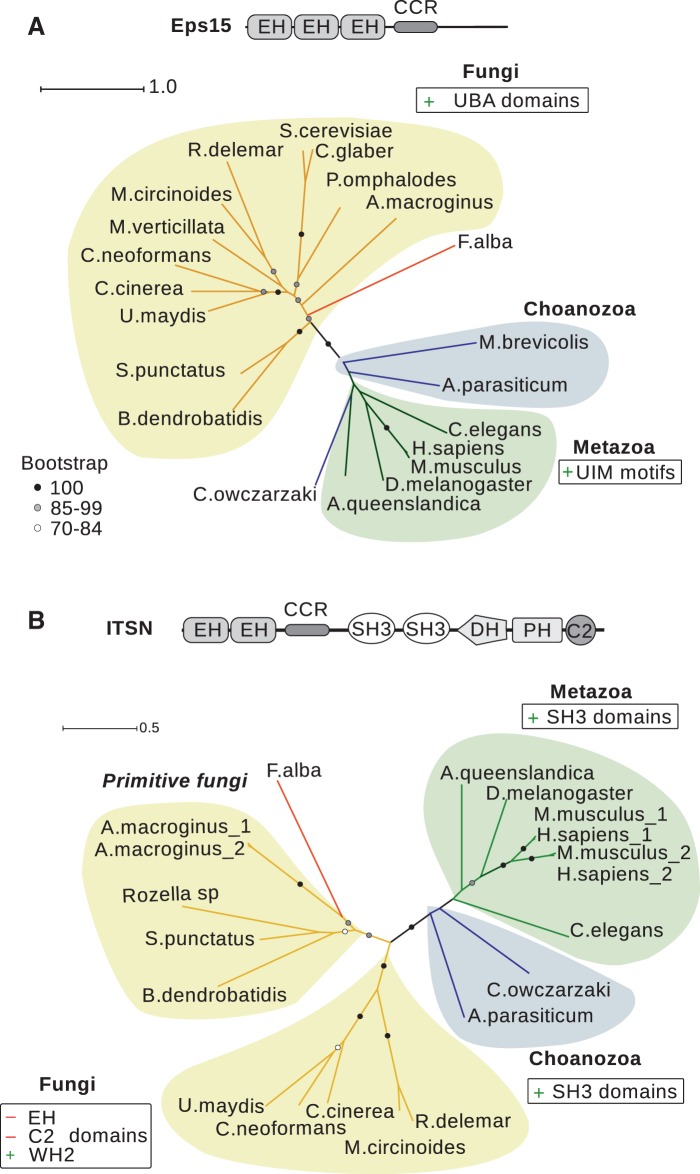
Unrooted phylogenetic trees demonstrate phylogenetic relationships between selected homologs of Eps15 (*A*) and ITSN (*B*). Circles indicate bootstrap support values >70 from 100 replicates. Scale bars show genetic distances. Domain organization of inferred ancestor proteins are indicated above each ML tree, and gain and loss of domains/motifs in major branches are shown.

Phylogenetic analysis of Eps15 homologs using the maximum likelihood (ML) method revealed two clusters of proteins corresponding to fungal and metazoan branches which differed in the type of ubiquitin-binding domains, ubiquitin-associated domain (UBA) or ubiquitin-interacting motif (UIM) (fig. 3*A*). Thus, the ability to recognize ubiquitinated proteins was acquired by Eps15 homologs independently in the fungal and metazoan branches as a result of convergent evolution suggesting an essential role of Eps15 in recognizing ubiquitinated proteins, particularly endocytic cargoes, both in fungi and metazoans.

Homologs of ITSN were clustered into three major clades according to the ML tree (fig. 3*B*). Two clades included fungi and animals with choanozoans. The third clade represented primitive fungi and the nucleariid *F. alba* that are considered closely related to the phylogenetic root of opisthokonts (Paps et al. 2013). The domain architecture of ITSN proteins found in the third clade was assumed as primeval for this protein. It includes all the typical domains of animal ITSN except for three additional SH3 domains gained in holozoans. Loss of various ITSN domains (the EH and C2 domains) in different fungal taxa (fig. 3*B*) and gain of a WASP-homology 2 domain domain involved in actin binding could reflect alterations of its role in an advanced fungal group. Among all ITSN proteins the DH-PH tandem implicated in regulation of the small GTPase Cdc42 activity is the most invariant part throughout opisthokonts, suggestive of its important role.

Thus, in opisthokonts conserved EEC proteins acquired additional functional regions for cargo recognition as well as for interactions with endocytic components, PM and actin cytoskeleton regulators.

### FCHO as a Regulator of the Core Endocytic Complex AP2

The third FEI component, the FCHO protein of the muniscin family, is composed of a membrane-bending Fer/Cip4 homology domain (FCH) domain and a protein-binding mu homology domain (muHD). This protein plays an essential role in initiation of CME by clustering AP2 (Henne et al. 2010) and contributing to its conformational switch and activation in animals (Hollopeter et al. 2014) The search for FCHO ancestors revealed that the FCH domain was not typical of bikonts, whereas in unikonts this domain was a structural part of multiple proteins (fig. 2). ML analysis of FCH domains produced a phylogenetic tree with low bootstrap support values (supplementary fig. S1*A*, Supplementary Material online). Poor differentiation of FCHO structures prevented us from defining phylogenetic relationships between various FCH domains. In contrast to the FCH domains, muHD domains were widely distributed throughout eukaryotes (fig. 2). In the phylogenetic tree, muHD domains formed distinctive clades that corresponded to AP1–AP5 complexes, COPI (coat protein I), and the muniscin family (supplementary fig. S1*B*, Supplementary Material online). In the latter, clade sequences of fungal and animal muniscins clustered with two muHD-containing proteins of Bikonts, the plant *Arabidopsis thaliana* (TML) and the excavate *Naegleria gruberi.* The role of the plant muniscin-like protein TML was reported in CME as a component of the plant-specific endocytic TPLATE complex (Gadeyne et al. 2014). Thus, based on structure–functional data, the muHD-containing protein found in excavates could be considered a predecessor of muniscins.

**Fig. 4.—:**
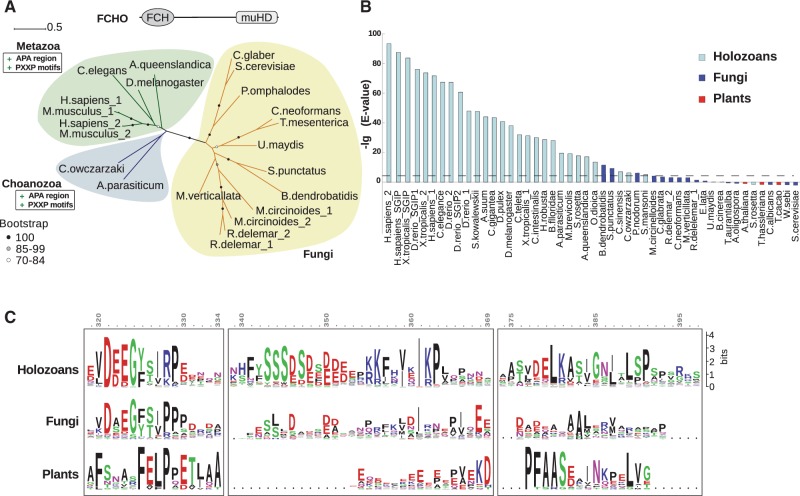
Evolution of FCHO homologs. (*A*) The ML tree shows the phylogenetic relations between FCHO proteins. Bootstrap values are indicated by circles, genetic distances are shown as scale bars. Domain architecture of the inferred ancestral domain organization of the FCHO protein is shown, and gain of functional regions is indicated. Numbers next to species name denote several paralogs within individual proteome. (*B*) Presence of APA regions in homologs of FCHO or related proteins according to *e*-values. The HMM profile was built based on APA sequences of muniscins, FCHO and SGIP proteins, of *Homo sapiens*, *Mus musculus*, and *Caenorhabditis elegans*. Negative logarithms of *e*-values are plotted on the diagram. The threshold corresponding to an *e*-value equal to 0.01 is shown as a dashed line. Colors of the bars represent taxonomy. (*C*) Central regions of the FCHO and related proteins were aligned. Conserved residues specific of each group are shown as logo. Numbers correspond to the positions of amino acid residues in the human FCHO2 protein.

Phylogenetic analyses of FCHO homologs demonstrated two clades in the ML tree that corresponded to holozoan and fungal branches (fig. 4*A*). We analyzed FCHO homologs for the presence of the AP2-activating (APA) region reported in animals. The HMM profile generated using APA motifs of human, mouse, and nematode FCHO homologs (Hollopeter et al. 2014) was used to search in proteomes of eukaryotes using HMMer (http://hmmer.janelia.org/, last accessed May 1, 2015). The data obtained demonstrated that sequences corresponding to the APA motif are unique of holozoan FCHO proteins, whereas fungal homologs as well as plant TML lack this region (fig. 4*B* and *C*). In primitive fungi, a conserved region of 15 amino acid residues similar to the beginning of the APA motif was found (fig. 4*C*); however, it was not sufficient for the function of APA (Umasankar et al. 2014). Thus, the functional APA region apparently was acquired in FCHO homologs of holozoans.

The APA region of FCHO facilitates the closed-open conformational switch of the AP2 complex (Hollopeter et al. 2014). We suggest that emergence of the APA motif in holozoans accompanied structural changes in AP2 that led to the closed conformation requiring a specific activator. A number of point mutations in the AP2 subunits each rendered the conformational switch of the complex less dependent on activation by the APA motif (Hollopeter et al. 2014). We asked whether such substitutions could be observed in AP2 subunits of fungi and other eukaryotes that lack the APA motif in FCHO. To probe this we used fungi as closest relatives of holozoans. Analysis of the group comprising all other eukaryotes except opisthokonts could answer the question of whether the presence/absence of amino acid substitutions in fungal AP2 was typical of all eukaryotes beyond holozoans.

As expected, most residues reported as essential for the closed AP2 conformation (Hollopeter et al. 2014) were conserved throughout eukaryotes (supplementary fig. S2 and table S5, Supplementary Material online). However, conserved residues in positions 195 (μ195) and 306 (μ306) of the mu subunit as well as 451 (α451) of the alpha subunit were distinct between holozoans and other eukaryotes. These differences resembled point mutations that impaired closed conformation of AP2 in worms (Hollopeter et al. 2014) and therefore attracted our attention. Other residues conserved in holozoans, μ290, μ298, μ378, and μ379, were variable in other eukaryotes.

**Fig. 5.—:**
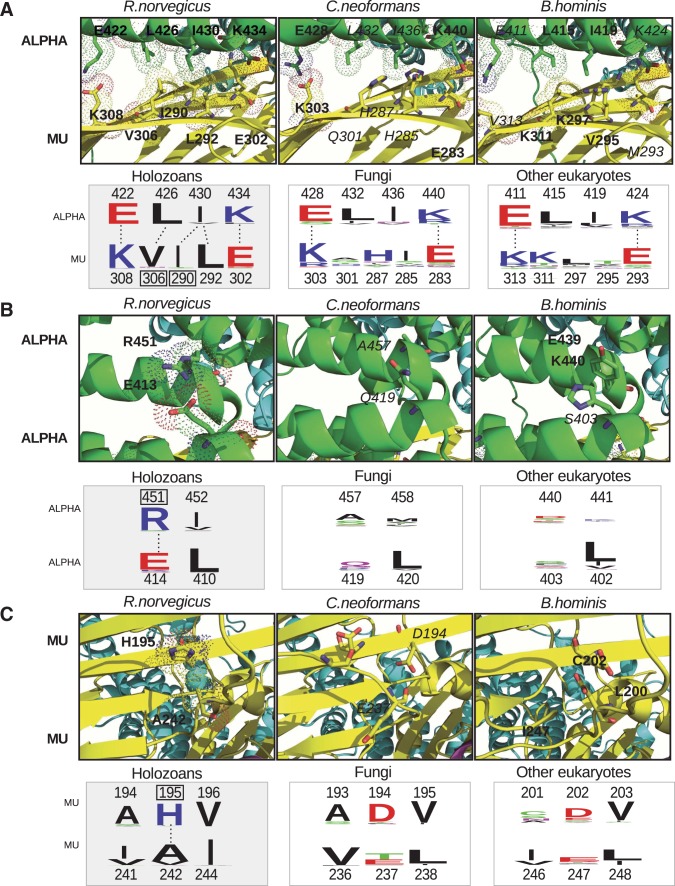
Amino acid residues supporting inter- and intramolecular contacts of AP2 subunits. Contacts within resolved AP2 structure of *Rattus norvegicus* (left panels), and generated models of *Cryptococcus cinerea* (middle panels) and *Blastocystis hominis* (right panels) involving residues essential for the closed conformation (Hollopeter et al. 2014) are shown. Interactions between alpha and mu (*A*), within alpha (*B*) and within mu (*C*) subunits are shown. Dots show deduced interacting residues (in bold); absence of dots indicates lack of contact (residues in italics). Schematic representation of contacts is shown in lower panels where lines indicate interactions. Levels of conservation of amino acids involved in the contacts are shown by logos. Numbers indicate positions of amino acid residues in AP2 subunits of *R. norvegicus*, *C. cinerea*, and *B. hominis* representing holozoans, fungi, and other eukaryotes, respectively. Positions of residues identified by mutational analysis in animals (Hollopeter et al. 2014) are framed on logos.

Structural superposition of the resolved *R. norvegicus* AP2 complex (4UQI) and the generated models of the fungal *Cr. cinerea* and chromalveolata *B. hominis* AP2 complexes in closed conformation (supplementary file S1, Supplementary Material online) was used to deduce contacts of residues μ306 (fig. 5*A*), α451 (fig. 5*B*), and μ195 (fig. 5*C*). The residues involved in the identified contacts were conserved in holozoans, whereas in fungi and other eukaryotes these residues were variable or distinct and contacts were hindered in AP2 complexes of *Cr. cinerea* and *B. hominis*.

The contact between the alpha and mu subunits (fig. 5*A*) is supported in rat AP2 by hydrophobic and electrostatic interactions that include residues conserved in holozoans, μ306 and μ290. In rat, AP2 hydrophobic association of the μV306/αL426 and μI290/αI430 pairs as well as electrostatic contacts of the μK308/αE422 and αK434/μE302 pairs were observed (fig. 5A, left panel). In fungi and other eukaryotes, the absence of hydrophobic interactions did not stabilize contacts between charged residues (fig. 5A, middle and right panels), and the residues in the identified positions were less conserved and distinct from those observed in holozoans.

Within the alpha subunit of rat AP2, the proximity of conserved αR451/αE414 residues provided juxtaposition of alpha helices (fig. 5B, left panel) in the closed AP2 conformation, whereas in the open state the distance between these residues increased. In fungi and other eukaryotes, residues in corresponding positions are not conserved (fig. 5B, middle and right panels) indicating a lack of this intramolecular contact.

Hydrophobic interaction of the proximally located conserved μH195/μA242 residues was observed within the mu subunit of rat AP2 (fig. 5C, left panel), whereas in other eukaryotes negatively charged residues in corresponding positions (fig. 5C, middle and right panels) apparently created repulsive forces.

Our data suggest that in holozoans, conservation of residues distinct from those in other eukaryotes contributed to the closed conformation of the AP2 complex that required a specific activator, the APA motif of the FCHO protein.

### Development of Cargo Selection during Evolution of CME

To couple various cargoes destined for internalization with the endocytic machinery, adaptor proteins such as the AP2 complex and specific sorting proteins are required (Traub 2009). Although the AP2 complex is ubiquitous in eukaryotes, different CLASPs were gained sequentially during evolution (fig. 1A). To investigate how the interface between the vesicle-forming machinery and cargoes evolved, we focused on the evolutionary history of known CLASPs. We analyzed the presence and conservation of features essential for CLASPs’ function: Cargo-binding regions and linear recognition motifs for endocytic interaction hubs and accessory proteins.

**Fig. 6.—:**
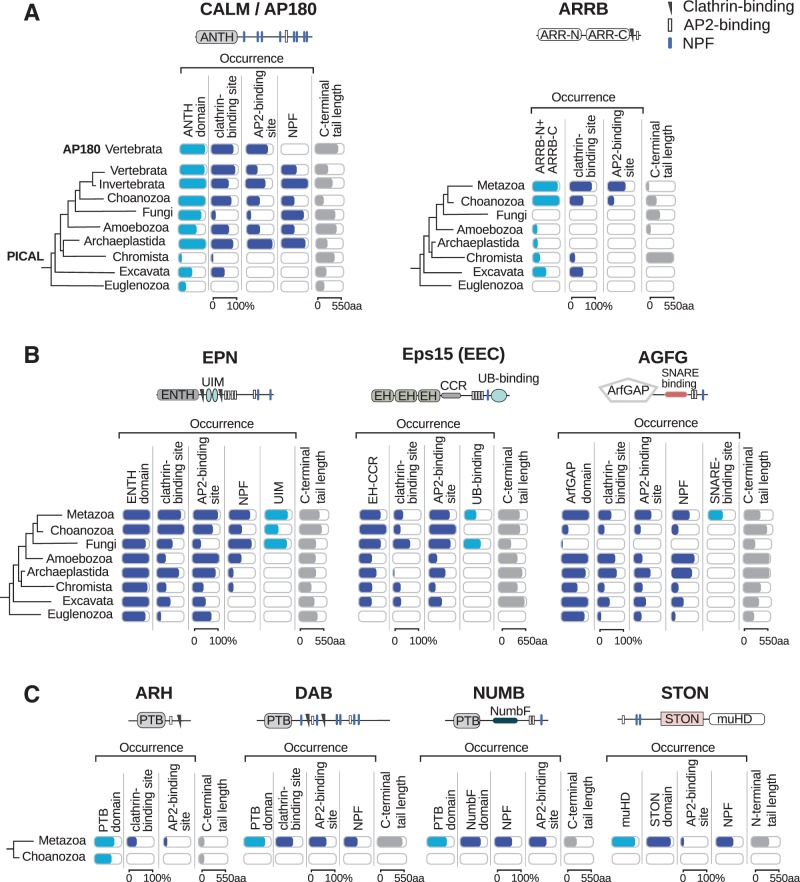
Origin of CLASPs. Conservation of domains, linear motifs, cargo-binding sites, and average length of tails of CLASPs are shown as bars. (*A*) CLASPs found in distant eukaryotic branches. (*B*) Endocytic accessory proteins that during evolution on the way to animals obtained cargo-binding sites. (*C*) CLASPs derived predominantly from signaling molecules. Homologs of the animal CLASPs identified were analyzed for the presence of domains, linear motifs, and the length of the tails. Occurrence of the domain/motif per taxon for a particular homolog reflects conservation of the feature. Cargo-binding sites are represented in light blue, domains and motifs in blue, and tails in gray. Structures of proteins typical of humans are shown above each panel. Phylogenetic relations of systematic groups are indicated on the left.

CALM and ARRB (arrestin beta) were found in most eukaryotic supergroups suggesting their presence in LECA (fig. 6*A*). The cargo-binding AP180 N-terminal homology domain of CALM was highly conserved across eukaryotes (fig. 6*A*, left panel) as well as residues (Miller et al. 2011) involved in soluble NSF attachment protein receptor (SNARE) binding (supplementary table S6, Supplementary Material online). CALM homologs of Euglenozoa, the earliest branch of eukaryotes, lacked clathrin- and AP2-binding motifs. Given that clathrin of euglenozoans is very distant from its animal homolog (supplementary table S1, Supplementary Material online), cargo sorting in this taxon could involve distinct interaction motifs and scenarios. Poor conservation of CALM was observed in chromists, whereas in other eukaryotic branches this protein became highly conserved and acquired additional contacts with the endocytic machinery due to gain of AP2-binding sites and NPFs. In vertebrates, the paralogous AP180 protein emerged (fig. 6*A*, left, upper row; supplementary fig. S3, Supplementary Material online) with localization restricted to neurons and specialized functions in synaptic vesicle recycling (Yao et al. 2002). Another sorting protein that was suggested to have emerged in LECA was ARRB; however, its decay or complete loss was observed in most eukaryotic branches except Excavata and Holozoa (fig. 6*A*, right). The AP2-binding motif typical of animal ARRBs that contributes to receptor-mediated endocytosis (Zhang et al. 1997) was found in holozoan ARRB homologs only. In spite of the lack of detectable ARRB homologs in certain taxa, G-protein coupled receptor (GPCRs) could be found across all eukaryotic branches (de Mendoza et al. 2014). Notable, in animals ARRBs mediate fast internalization of GPCRs, while slow uptake mechanism independent of ARRBs and clathrin was also reported here (Koppen and Van Jakobs 2004). Presumably, in eukaryotic branches lacking ARRBs endocytosis of GPCRs is mediated via alternative internalization routes.

The accessory proteins EPN, Eps15 (EEC beyond opisthokonts), and AGFG were highly conserved endocytic components; however, in early branched eukaryotes they did not contain cargo-binding regions (fig. 6*B*). The homologs of EPN and Eps15 gained the ability to recognize ubiquitinated cargoes via UBA and UIM regions in opisthokonts. A specific region to have recruited SNAREs (Pryor et al. 2008) has been found in AGFG homologs since metazoans (fig. 6*B*, right panel; supplementary table S7, Supplementary Material online). The increase in the number of linear motifs in these CLASPs on the way to animals (supplementary table S8, Supplementary Material online) was documented.

Sorting proteins that emerged in metazoans were built on the basis of either cargo-binding phosphotyrosine-binding domain (PTB) domains of signaling proteins (such as for DAB [disabled homolog] and NUMB [protein numb homolog]) or the muHD domain of the AP2 mu subunit (such as for STON) (fig. 6C). These proteins immediately possessed regions necessary for cargo recognition and interaction with endocytic proteins. Autosomal recessive hypercholesterolemia homologs (ARH) had poorly conserved binding sites for clathrin and AP2 in animals and completely lacked these motifs in the early branch, the choanozoans.

Thus, LECA possessed sorting proteins essential for engagement of cargoes necessary for vesicle fusion and signal transduction processes. Starting from opisthokonts, endocytic accessory proteins gained ability of cargo recruitment via acquisition of cargo-binding regions. The most evolutionary recent CLASPs specific of metazoans were based on domains typical of signaling pathways.

### Emergence of DNM as a Central Regulator of Late Endocytic Stages

The late stage of CME requires constriction of the vesicle neck, recruitment of the DNM GTPase, and coordinated polymerization of actin to promote vesicle scission (Lundmark and Carlsson 2004; Shin et al. 2008).

**Fig. 7.—:**
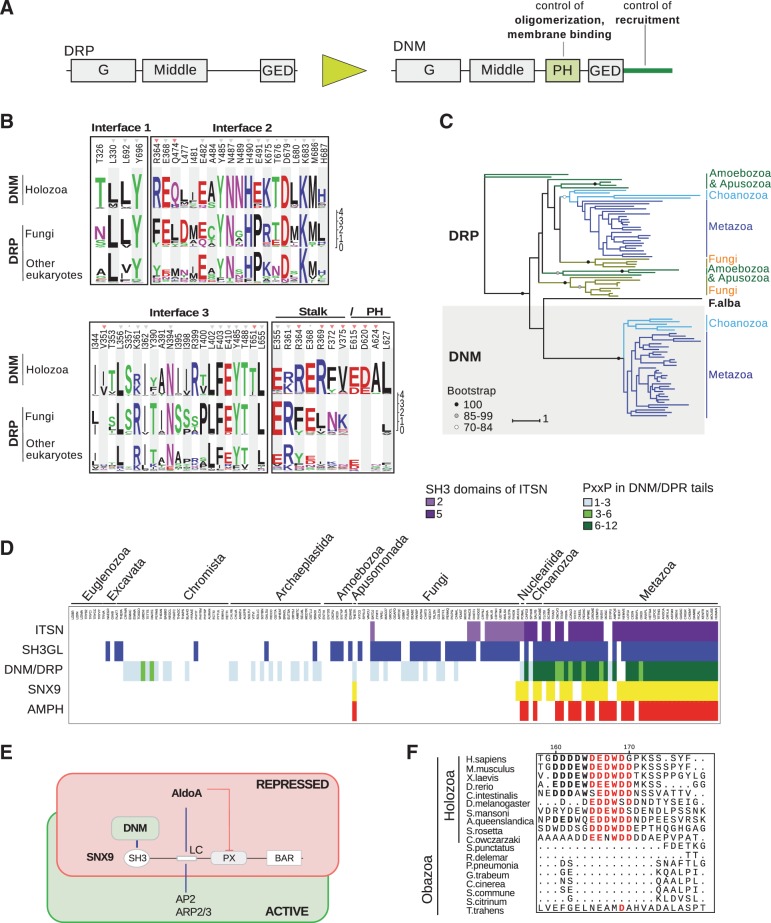
Evolution of DNM and ways of its regulation. (*A*) Schematic representation of DRP and DNM domain architectures; distinct features are highlighted in green, their known roles are indicated above. (*B*) Comparison of interaction interfaces involved in oligomerization within DRP and DNM molecules. Homologs of DNM (supplementary table S1, Supplementary Material online) and DRP (supplementary table S2, Supplementary Material online) were aligned, and the residues comprising interfaces (Reubold et al. 2015) were obtained. The conservation of these residues is shown as logo. Gray triangles above denote conserved residues in DRP and DNM across eukaryotes, gray dots show similar residues within opisthokonts, and red triangles denote specific conserved residues in DNM across holozoans. (*C*) ML tree shows the phylogenetic relations of the DRP and DNM proteins in selected representatives of unikonts. Circles represent bootstrap support values, scale bars indicate genetic distances. (*D*) The diagram shows the distribution of proteins and their functional regions in the proteomes indicated above. (*E*) Schematic representation of the regulation of DNM recruitment by SNX9 and aldolase. (*F*) Multiple sequence alignment of the fragment within the linker of SNX9 homologs responsible for AldoA binding. The conserved residues across holozoans are highlighted in red, and across animals in bold.

According to our results (supplementary table S1, Supplementary Material online) and previously published data (Liu et al. 2012), homologs of DNM were found only in holozoans. In other eukaryotic branches dynamin-related proteins (DRPs) lacking the pleckstrin homology (PH) domain and prolin-rich C-terminal tail were found (fig. 7*A* and supplementary table S2, Supplementary Material online). We followed transformation of DRP to DNM analyzing its key functional regions. The three crucial interfaces for dimer and tetramer formation (Reubold et al. 2015) were highly similar in DRP and DNM across eukaryotes (fig. 7*B* and supplementary table S9, Supplementary Material online). However, DNM had a specific interaction interface between the PH domain and middle domain (MD) in contrast to DRPs (fig. 7*B*). Using ML analysis, a putative transitional DRP-to-DNM protein, DNL1A (fig. 7*C*), with distinctive MD was identified among three DRPs of *F. alba* (supplementary fig. S4*A*). The other domains, GTPase and GTPase effector domain, of identified nucleariid protein were typical of DRP (supplementary fig. S4*B* and *C*), suggesting that transformation of DRP to DNM was initiated in the MD.

Next, we analyzed the C-terminal tails of DNM and DRP as the major platform of DNM recruitment to CCPs (Sundborger and Hinshaw 2014). Various DRPs of eukaryotes possessed a C-terminal tail; however, it was not conserved and lacked multiple PxxP motifs present in classical DNM (fig. 7D and supplementary table S10, Supplementary Material online). Within the transitional DRP-to-DNM protein of *F. alba*, a long C-terminus with PxxP motifs necessary for SH3 domain interactions was documented. The distribution across eukaryotes of SH3-containing proteins, SNX9 and AMPH, that are important recruiters of DNM (Sundborger and Hinshaw 2014), coincided with the presence of PxxP motifs in the DNM/DRP tails (fig. 7D and supplementary table S10, Supplementary Material online). These proteins were first found in the apusomonad *T. trahens* that belongs to a group that branched prior to Opisthokonta (supplementary tables S1 and S4, Supplementary Material online). Within opisthokonts, SNX9 and AMPH as well as DRP with a proline-enriched C-terminus were poorly conserved or lost in fungi. However, these SH3 domain proteins together with their target motifs in DNM became conserved in holozoans. Noteworthy, another SH3-containing regulator of this GTPase, the ITSN protein, underwent expansion of its SH3 domains from two to five in this taxon (fig. 7*D*).

In the cytosol, DNM persists in a repressed state in complex with SNX9 and the glycolytic enzyme aldolase, AldoA (fig. 7*E*). Aldolase prevents binding of SNX9 to the PM via interaction with the Phox homologous domain (Lundmark and Carlsson 2004). As SNX9 was poorly presented in the reference fungal proteomes used in this study, exhaustive search for SNX9 homologs through the UniProt and NCBI databases was performed. A conserved motif with negatively charged and hydrophobic residues required for binding to aldolase (Rangarajan et al. 2010) was found in SNX9 of holozoans but not in fungi or *T. trahens* (fig. 7F and supplementary fig. S5, Supplementary Material online). A high level of aldolase conservation as well as its residues involved in SNX9 binding (Rangarajan et al. 2010) was observed throughout eukaryotes (supplementary table S11, Supplementary Material online).

Thus, multiple concurrent changes contributed to the emergence of novel mechanism of vesicle fission based on the mechanochemical GTPase DNM and included regulators of membrane curvature and actin polymerization. The emergence of DNM from DRP occurred in several steps: The first transformation apparently was initiated in the MD, then the C-terminus gained multiple linear motifs for SH3 domain binding, and the PH domain and corresponding interaction interface emerged. The distribution and conservation of SH3-containing interactors of DNM overlap with the presence of PxxP motifs within the C-terminal tail of the GTPase. Thus, apparently, the mechanism of negative regulation and bringing DNM to endocytic sites via the SH3-containing recruiters was accomplished in holozoans.

## Discussion

The complexity of modern organisms is a result of long-term evolution. All the features of living creatures are based on sophisticated interplay of events at the molecular level. Yet, how was the molecular basis of known processes gained, which steps did it take, and what was its relative timing? Trying to answer these questions in the field of endocytosis, we followed transformation of the CME machinery during the course of eukaryotic evolution. The most ancient core endocytic proteins typical of most eukaryotes were defined as well as gains of novel components together with their links to existing components. The results showed that CME underwent many-sided changes affecting early and late stages of CCV formation, and the way of loading the endocytic cargoes.

The analysis revealed that LECA possessed a subset of 22 of 35 proteins crucial for animal CME that apparently were sufficient to form CCVs. The core CME proteins clathrin and AP2, as well as proteins involved in membrane deformation, clathrin assembly, recruitment of the actin cytoskeleton, and vesicle uncoating were found in major eukaryotic branches. These data are in line with previous studies that reported the ubiquitous nature of CME in eukaryotes (Field et al. 2007).

**Fig. 8.—:**
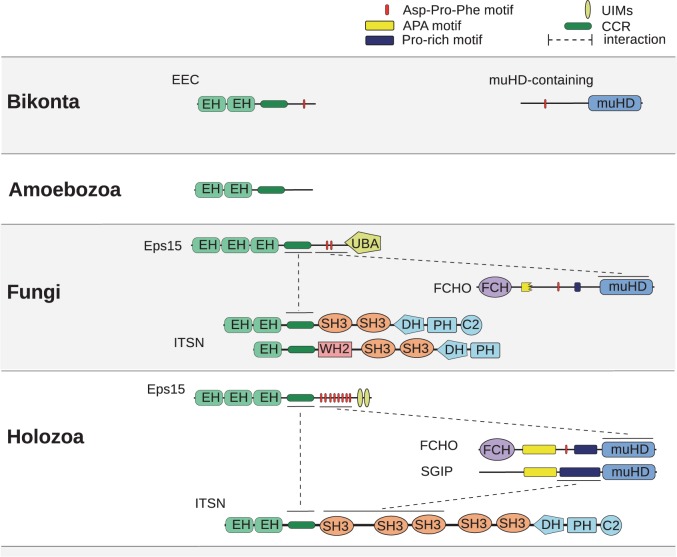
Domain architecture and interactions of FEI components and related proteins of various taxa. Known protein interactions are shown as dashed lines. Homologs of ITSN are lost in ascomycetes; in vertebrates, FEI components are represented by two paralogous proteins each; SGIP represents a vertebrate-specific member of the muniscin protein family with expression restricted to the brain.

One of the early gains of multiple endocytic proteins occurred in opisthokonts. Three functionally linked proteins (FCHO, Eps15, and ITSN) were derived from conserved ancestral molecules. The proteins mentioned constitute the FEI functional complex, needed for early and intermediate steps of CME in animals. Ancestors of the FEI components, the EEC and muHD-containing proteins, were found in eukaryotic branches beyond opisthokonts, in particular in excavate *N. gruberi* that belongs to the earliest branch of extant eukaryotes (Cavalier-Smith et al. 2014). Moreover, proteins with the same domain organization were found in plants within the endocytic TPLATE complex (Gadeyne et al. 2014). All EEC proteins studied to date, in particular the plant AtEH1/2 (Gadeyne et al. 2014), the fungal Ede1, End3p, and Pan1p (Weinberg and Drubin 2012), as well as the animal Eps15, ITSN, and Reps (Cullis et al. 2002) are involved in CME. The high level of conservation of EEC proteins across eukaryotes and their low number in proteomes indicated a specific role of EEC proteins in the mechanism of CME in eukaryotes. We suggest that Eps15 and ITSN are opisthokont-specific variants of conserved EEC proteins. Ubiquitin-binding domains were added to the EEC C-terminus to form the Eps15 protein, while multiple SH3 domains and the DH-PH-C2 terminus were combined with EEC within ITSN. Conserved in eukaryotes the muHD-containing protein gained an FCH domain resulting in the FCHO protein. Modules responsible for interaction of EEC-containing proteins with FCHO homologs within the FEI proteins have been found in these proteins since their emergence. In opisthokonts, FCHO, Eps15, and ITSN underwent extensive lineage-specific transformations (fig. 8). Despite variability of structures the FEI proteins play important role in early stages of CME both in fungi and animals (Henne et al. 2010; Mayers et al. 2013; Boeke et al. 2014.

Addition of novel components to the CME machinery led to modifications of the endocytic PIN. We propose that FEI functional network became the third endocytic hub along with AP2 and clathrin that integrate the vesicle-forming machinery and CLASPs recruiting specific cargoes to the nascent pits. The existence of functionally distinct groups relies on the universal role of accessory proteins in vesicle formation and on the specific function of CLASPs in the selection of a certain cargo. Both AP2 complex and FEI proteins are required for initial steps of CME (Henne et al. 2010; Kirchhausen et al. 2014). The FEI functional complex could function in cooperation with AP2 or more intriguingly could mediate alternative scenarios of AP2-independent internalization. The available data support this hypothesis. CME occurs without AP2 in intact cells as well as upon depletion of the adaptor complex (Motley et al. 2003; Mettlen et al. 2009). Moreover, efficient internalization of integrin β1 cargo via the complex of DAB2 and Eps15/ITSN proteins occurred independently of AP2 (Teckchandani et al. 2012). Although the stable complex of the AP2 subunits was substantially documented (Kelly et al. 2014), the possibility of existence of the preassembled FEI complex was not established. The structure of the FEI functional network and stoichiometry of its components at the endocytic site remains unresolved; however, organization of this network could be quite complex regarding that both Eps15 and ITSN are able to form oligomers (Tebar et al. 1997; Wong et al. 2012).

Involvement of animal AP2 in CME is controlled by the FEI proteins. The latter recruits AP2 to the PM (Henne et al. 2010) and shifts the adaptor complex to an open (active) conformation (Hollopeter et al. 2014; Umasankar et al. 2014). The APA region within FCHO critical for the activation of AP2 emerged in holozoans. Concurrent structural changes in AP2 were documented that contributed to its closed state requiring an activator. The alpha and mu subunits of AP2 gained conserved residues responsible for contacts within and between subunits necessary for the closed conformation. In other eukaryotes corresponding residues were not conserved or distinct, indicating the lack of intra- and intersubunit contacts or the use of a different mechanism. Thus, the conformation of AP2 and therefore its functional activity became dependent on the FEI functional complex in holozoans.

CME gradually underwent integration with systems evolving on the way to animals, in particular signal transduction and cellular communication (Ben-Shlomo et al. 2003; Zaidel-Bar 2009). To provide noncompetitive internalization of numerous membrane proteins, specific sorting proteins were gained. CLASPs that recognize proteins required for the traffic of vesicles (SNAREs) and ancient signaling molecules (GPCRs) have been present since LECA. These are CALM and ARRB. Furthermore, during the period from the first opisthokonts to animals, new sorting proteins were derived from endocytic and signaling proteins via acquisition of cargo-binding regions and short motifs for recruitment to the sites of CME. The general evolutionary trend observed for the majority of CLASPs was acquisition of novel linear motifs and/or increase in their number. This could affect specificity and affinity of interactions within endocytic PIN as well as provide avidity-based complex assembly (Olesen et al. 2008). It is noteworthy that known cargoes for PTB-containing CLASPs typical for metazoans are restricted to molecules involved in signaling pathways and cell contacts (Traub 2009). It is expected that the current list of known CLASPs as well as their ligands is far from complete and progress in the investigation of animal CME *in vivo* will reveal new components of cargo selection and recruitment (Schmid et al. 2014).

We assume that the mechanism of deep invagination of the endocytic membrane and subsequent vesicle scission also underwent transformation on the way to animals. The critical set of components involving DNM and its positive and negative regulators were observed since holozoans. DNM was derived from DRP via several major innovations. The PH domain that controls oligomerization and membrane association was gained in holozoans. Another feature specific for DNM is the C-terminal tail enriched in PxxP motifs. We suggest that emergence of linear motifs in SNX9 (LC) and DNM (PxxP) accomplished the formation of the regulatory complex. Other components, SNX9 and AMPH, were gained earlier but became conserved in holozoans concomintantly with acquisition of linear motifs in DNM. This assumption is in line with the notion that linear motifs are important switchers of evolution of molecular complexes and interaction networks (Neduva and Russell 2005).

Polymerization of actin was shown to precede vesicle fission in animal cells (Merrifield et al. 2005). Two essential regulators of actin dynamics were identified at the sites of vesicle formation. Conserved in eukaryotes, the ARP2/3 (actin-related protein 2/3 complex) complex that is responsible for the formation of the branched actin network could be recruited to CCPs via interaction with the LC region of SNX9 in holozoans. In addition, holozoans acquired the actin regulator CTTN that accumulates in CCPs before fission at the same time as DNM (Sebé-Pedrós et al. 2011), apparently due to interaction with the enzyme via the SH3 domain. Emergence of the regulatory complex engaging DNM in holozoans is a good example of how emergence of linear motif-based interactions contributed to cooperation between systems responsible for membrane deformation-fission (SNX9-DNM, AMPH-DNM) and actin cytoskeleton polymerization (SNX9-CTTN, DNM-CTTN).

Recently, DNM was suggested to be a central component of the CME checkpoint in mammals capable of defining abortive and productive CCPs. The prolin-enriched C-terminus of DNM mediated numerous decisive interactions with SH3 domain-containing proteins capable of sensing membrane curvature, the presence of coat protein and cargo (Loerke et al. 2009). Formation of the molecular basis for the CME restriction point was accomplished in holozoans upon emergence of numerous PxxP motifs within DNM disordered C-terminus.

One of the last prominent changes of the CME machinery described was the generation of multiple paralogs in metazoans. Thus, 60% of the endocytic components studied were presented as protein families in vertebrates. Paralogs are often characterized by distinct expression profiles in tissues (DNM1/DNM2/DNM3, SYNJ1/SYNJ2, WAS/WASL, CLTA/CLTB, AMPH1/BIN1, FCHO1/FCHO2, CALM/AP180, etc.) or could differ in cellular localization (NECAP1/NECAP2, SNX9/SNX18/SNX33). However, in most cases the precise functional role of paralogous proteins remains so far unresolved. Paralogs specialized in different tissues or cellular compartments could reflect the need for fine-tuned specific regulation developed by endomembrane system during its evolution.

**Fig. 9.—:**
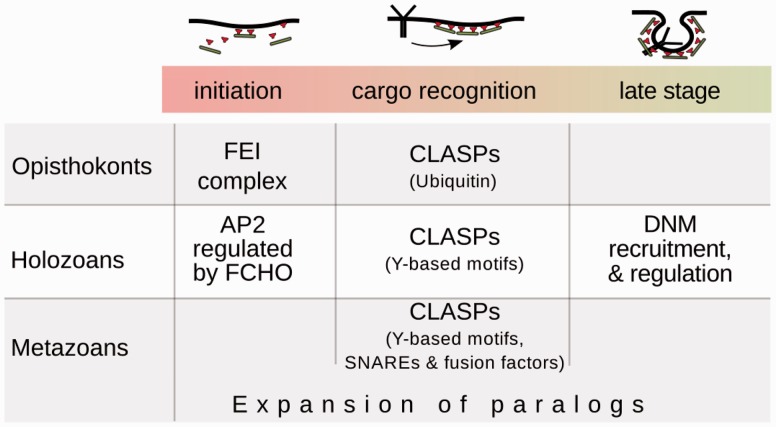
The major documented modifications of CME. During eukaryotic evolution addition to the CME machinery of components with novel features was observed as well as structure–functional adjustments of emerged and pre-existing components. A novel organizing center for endocytic components was gained in opisthokonts and could contribute to the efficiency of endocytic internalization. The FEI functional network became deeply integrated in the endocytic interaction network and was proposed as third interaction hub together with AP2 and clathrin. Additional mechanisms of fidelity control emerged in CME of holozoans apparently due to interconnection of endocytosis with evolving signaling pathways. In holozoans, activation of the AP2 complex during the early stage of CME became dependent on FEI, in particular interaction with the FCHO protein. Here the CME checkpoint emerged based on multiple interactions of the fission enzyme DNM with the SH3 domain accessory proteins. The emergence of DNM in holozoans as well as the mechanism of its recruitment and negative regulation resulted in changes in late CME stages. Expansion of PM receptor families and adhesion molecules essential for cellular integration and communication was accompanied by acquisition of various sorting proteins. CLASPs capable of recognizing specific sorting motifs could provide noncompetitive internalization of different cargoes. Within metazoans CME could undergo adaptation for cell type–specific functions, in particular via massive paralogous expansion of endocytic genes in vertebrates. Many redundant interactions of paralogous proteins could also contribute to the fault tolerance and robustness of the system.

The major documented alterations in CME are summarized in figure 9. Current analysis evidenced that animals together with sister group choanozoans have distinct elaborate mechanisms to control CME. More strictly regulated CME capable of internalizing multiple cargoes including molecules of adhesion and cell-to-cell contacts together with elaborate signaling could have impact on the emergence of multicellularity in animals (Sigismund et al. 2012). Notably, many systems of animal cells were formed in holozoans similarly to features that control fidelity of CME. For instance, regulation of biosynthesis (Brown et al. 2008), gene expression (Sebé-Pedrós et al. 2011), and signalling (Suga et al. 2014) typical of animals was gained in the common ancestor of holozoans. The data show essential differences between molecular machineries involved in CME in animals and widely exploited model of intracellular traffic, yeasts. Evolutionary analysis of endocytosis could be useful for interpretation of results and explain experimental discrepancies of investigations of CME using different model organisms. Moreover, it can predict specific features of endocytic internalization in certain taxa.

Our better understanding of the evolution of CME is currently limited by the lack of data for the period between the first and the LECAs that remains largely a black box for investigations. Another constraint is poor structure-to-function and sequence data for under-represented groups, that is, choanozoans, apusomonads, and others. The lack in certain species or taxonomic groups of CME components that are homologous to those specific of animals could suggest alternative scenarios of endocytosis. The function of a missing homolog could either be performed by a highly related protein as reported for the beta subunit of AP2 (Thomas Sosa et al. 2012) or a CME mechanism could be modified to an extent that does not require function of the homolog mentioned. These possibilities could suggest further directions to study endocytic mechanisms in eukaryotes.

## Supplementary Material

Supplementary tables S1–S11, figures S1–S5, and file S1 are available at *Genome Biology and Evolution online* (http://www.gbe.oxfordjournals.org/).

Supplementary Data
