# Supplementary material for: Evolutionary Changes on the Way to Clathrin-Mediated Endocytosis in Animals
Source: Genome Biol Evol. 2016 Feb 12;8(3):588–606. doi: 10.1093/gbe/evw028 (PMC4824007; doi:10.1093/gbe/evw028)
Supplement: Supplementary Data [file supp_8_3_588__index.html]

Evolutionary Changes on the Way to Clathrin-Mediated Endocytosis in Animals — Supplementary Data 

# Evolutionary Changes on the Way to Clathrin-Mediated Endocytosis in Animals

## Supplementary Data

files

- Supplementary Data - zip file
