# Supplementary material for: Evolutionary Changes on the Way to Clathrin-Mediated Endocytosis in Animals
Source: Genome Biol Evol. 2016 Feb 12;8(3):588–606. doi: 10.1093/gbe/evw028 (PMC4824007; doi:10.1093/gbe/evw028)
Supplement: Supplementary Data [file supp_evw028_suppl_data.zip › Supplementary Figures.pdf]

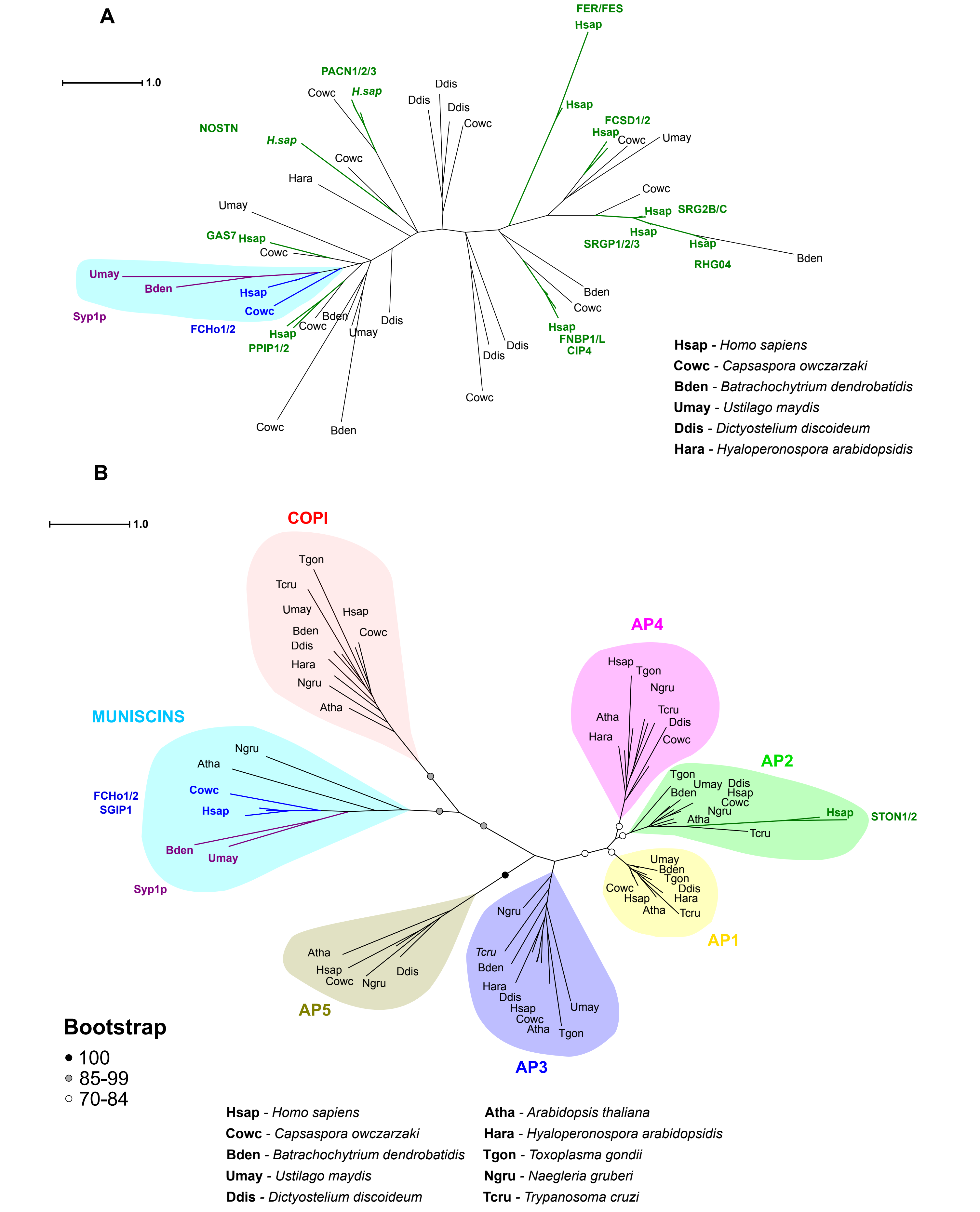

S1 Supplementary Figure. Phylogenetic relations between the FCH (A) and muHD (B) domains found in proteins of selected representatives of major eukaryotic branches.The shade of the circles represent bootstrap support values >70. Bars denote genetic distance. Human proteins in (A) are highlighted in green.

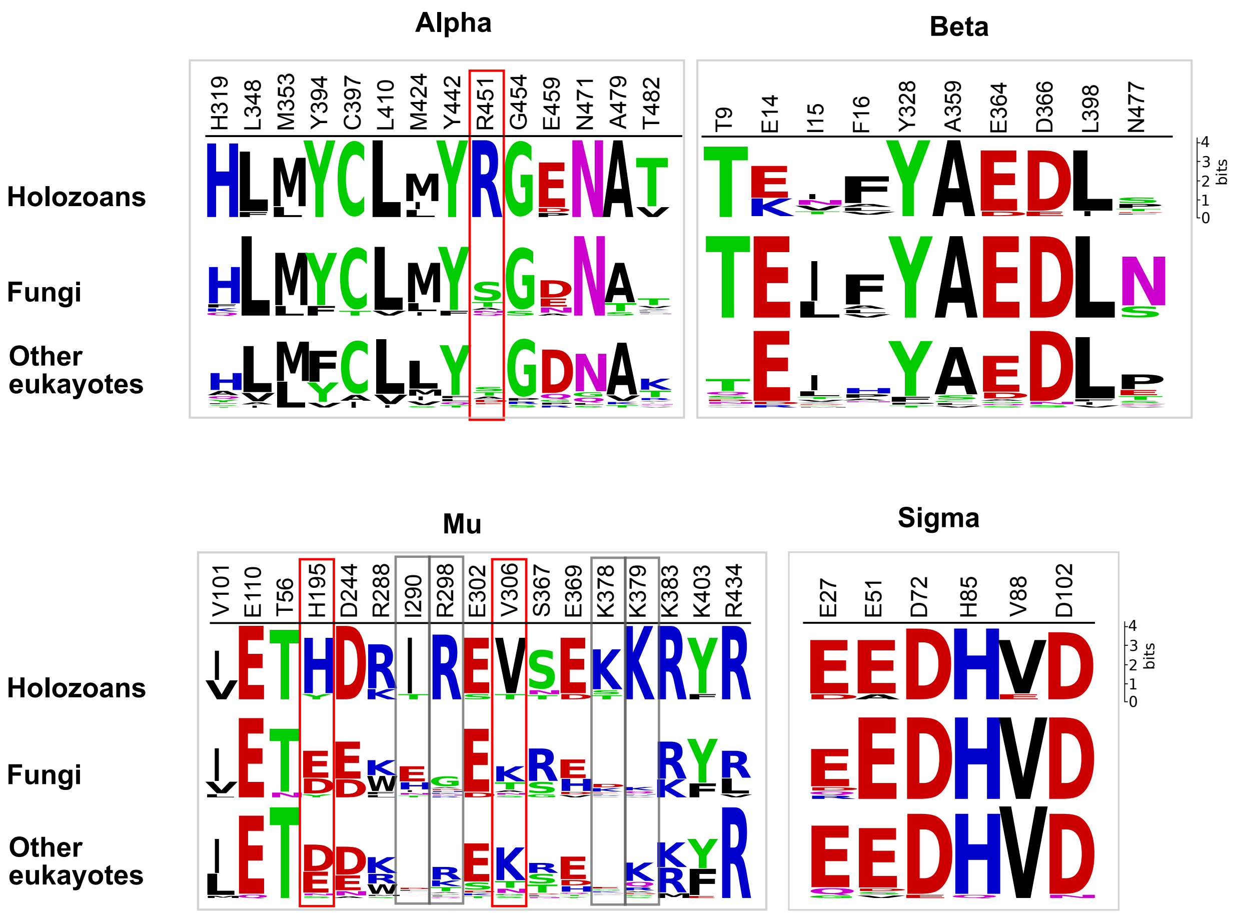

S2 Supplementary Figure. Conservation of residues important for closed conformation of AP2 (Hollopeter et al. 2014) in holozoans and corresponding residues of fungi and the rest of the eukaryotes. AP2 subunits were aligned by Clustal Omega, the corresponding residues in specific positions were obtained using Ugene, their conservation is shown as logo. Specific residues of holozoans are framed. Numbers above logos specify position corresponding to rat AP2 subunits. The list of organisms used is mentioned in Supplementary Table S1.

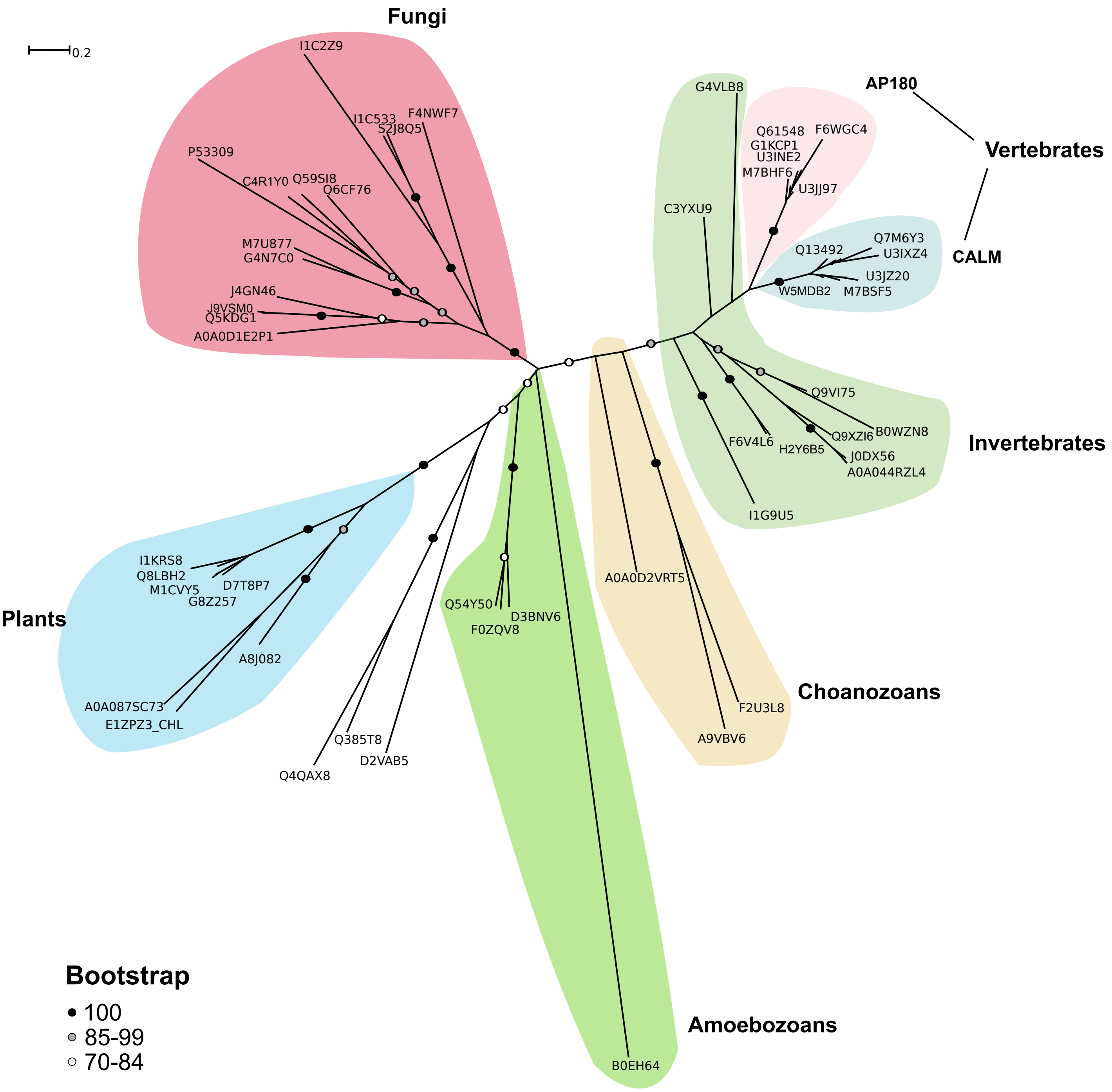

S3 Supplementary Figure. Phylogenetic relations between CALM and AP180 homologs. ML tree was built using RaxML; alignment was obtained by Clustal Omega. Corresponding protein sequences were obtained from UniProt according to Supplementary Table S1.

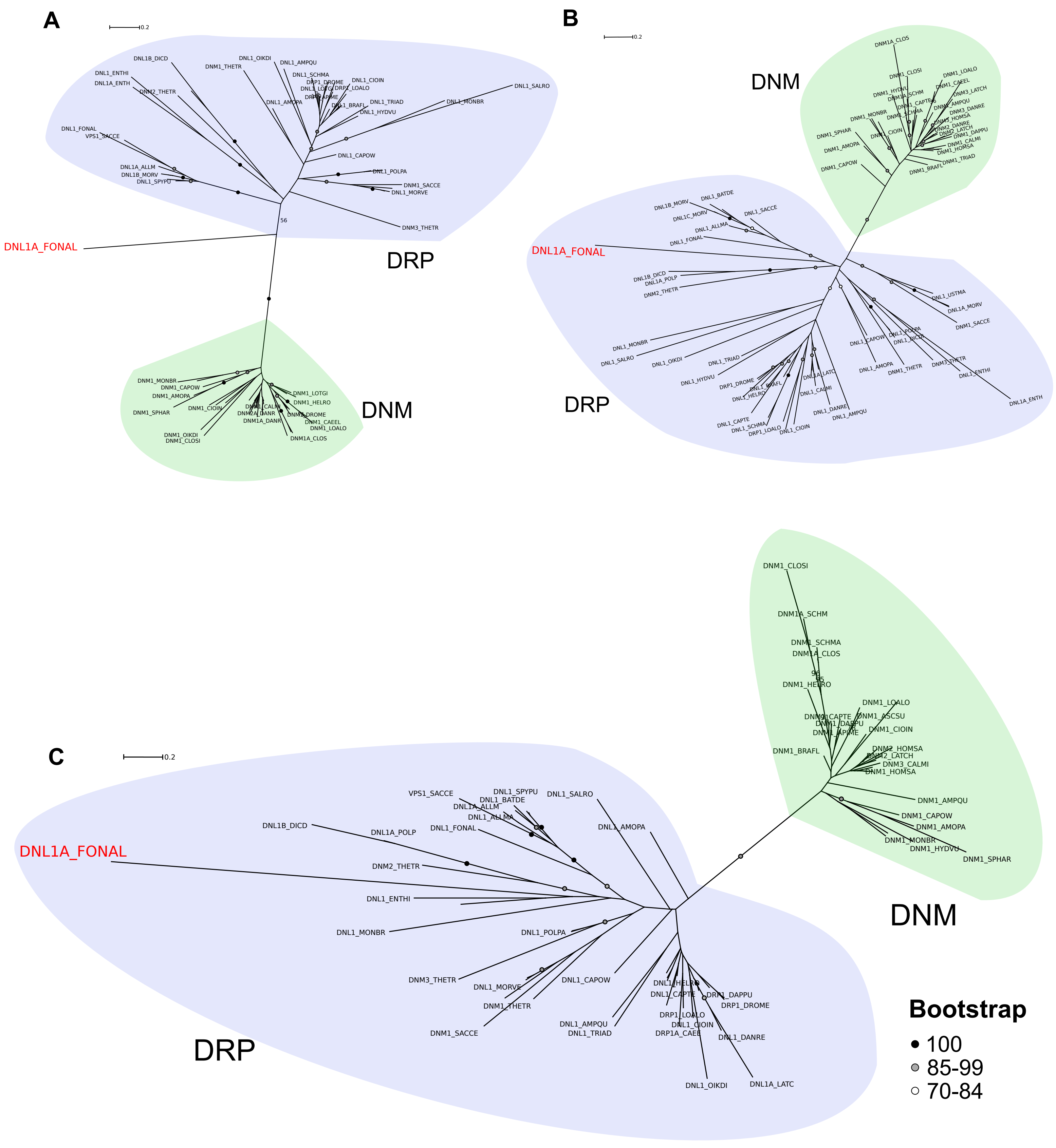

S4 Supplementary Figure. Phylogenetic relations of respective domains of DNM and DRPs. (A) Middle domains; (B) GTPase domains; (C) GED domains. Putative DRP-to-DNM transitional form of *F. alba*, DNL1A , is highlighted by red.

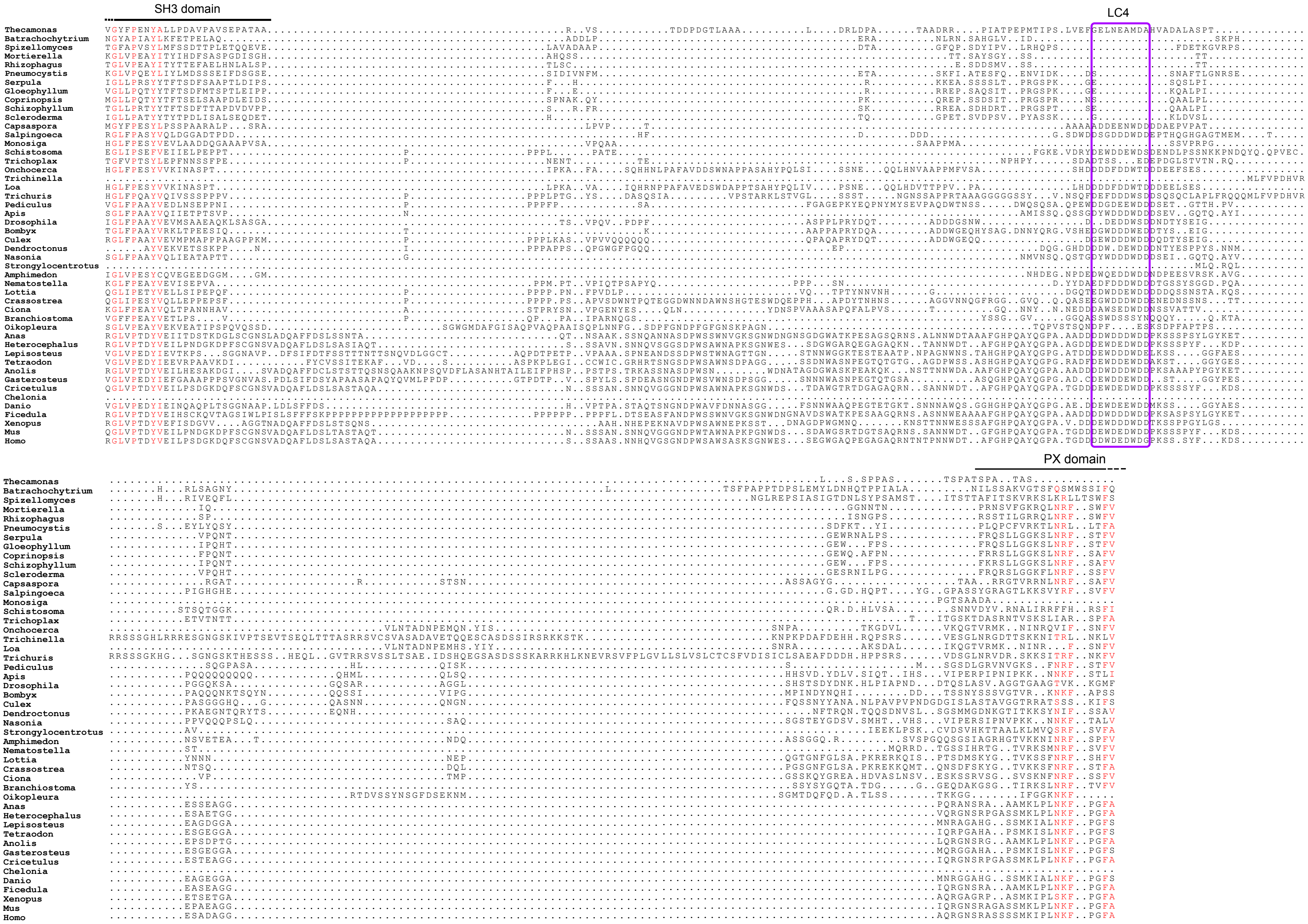

S5 Supplementary Figure. Multiple sequence alignment of the linker of SNX9 homologs. The end of the SH3 domain and the beginning of the PX domains are indicated, and the residues of the LC region are framed.
